# Supplementary material for: Effectiveness and Safety of Ayurvedic Medicines in Type 2 Diabetes Mellitus Management: A Systematic Review and Meta-Analysis
Source: Front Pharmacol. 2022 Jun 8;13:821810. doi: 10.3389/fphar.2022.821810 (PMC9213670; doi:10.3389/fphar.2022.821810)
Supplement: Supplementary file 1 [file DataSheet1.zip › Supplementary Material/Forest and Funnel Plots/Nigella sativa/PPBG.pdf]

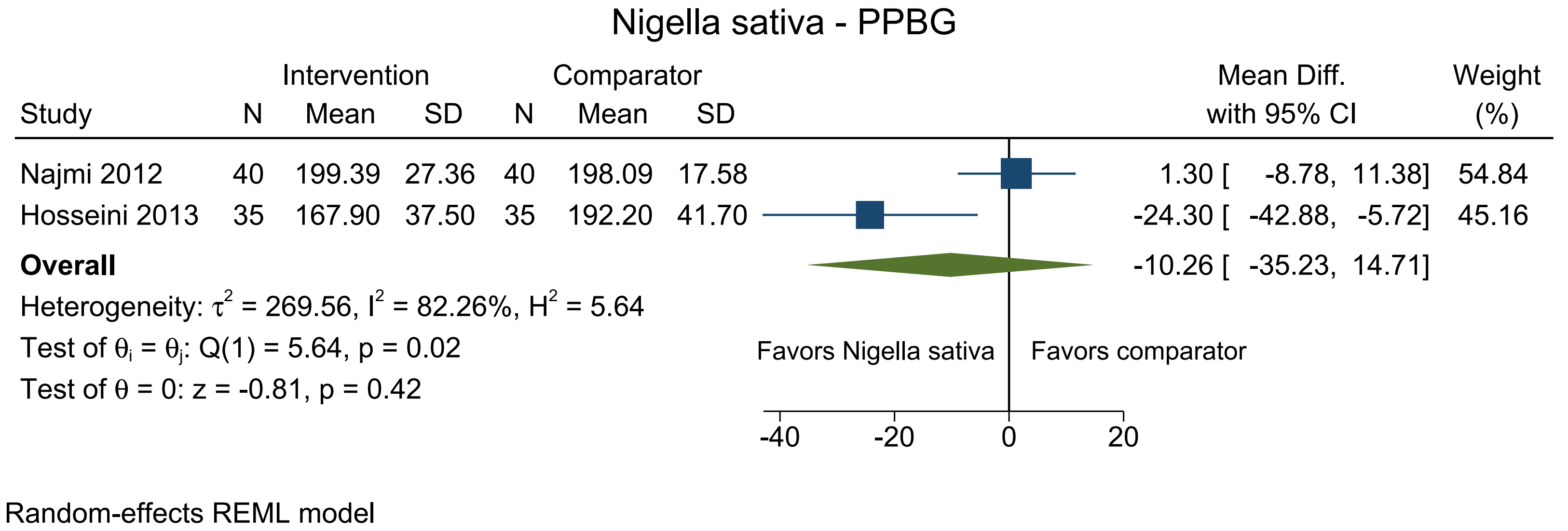
Nigella sativa - PPBG
Intervention
Comparator
Study
N
Mean
SD
N
Mean
SD
Mean Diff.
with 95% CI
Weight
(%)
Najmi 2012 40 199.39 27.36 40 198.09 17.58
1.30 [-8.78, 11.38] 54.84
Hosseini 2013 35 167.90 37.50 35 192.20 41.70
-24.30 [-42.88, -5.72] 45.16
Overall
-10.26 [-35.23, 14.71]
Heterogeneity: τ² = 269.56, I² = 82.26%, H² = 5.64
Test of θi = θj: Q(1) = 5.64, p = 0.02
Test of θ = 0: z = -0.81, p = 0.42
Favors Nigella sativa
Favors comparator
-40
-20
0
20
Random-effects REML model
